# Clinical Presentation, Convalescence, and Relapse of Rocky Mountain Spotted Fever in Dogs Experimentally Infected via Tick Bite

**DOI:** 10.1371/journal.pone.0115105

**Published:** 2014-12-26

**Authors:** Michael L. Levin, Lindsay F. Killmaster, Galina E. Zemtsova, Jana M. Ritter, Gregory Langham

**Affiliations:** 1 Rickettsial Zoonoses Branch, Centers for Disease Control and Prevention, Atlanta, Georgia, United States of America; 2 Infectious Diseases Pathology Branch, Centers for Disease Control and Prevention, Atlanta, Georgia, United States of America; 3 Animal Resources Branch, Centers for Disease Control and Prevention, Atlanta, Georgia, United States of America; The Johns Hopkins University School of Medicine, United States of America

## Abstract

Rocky Mountain spotted fever (RMSF) is a tick-borne disease caused by *R. rickettsii* in North and South America. Domestic dogs are susceptible to infection and canine RMSF can be fatal without appropriate treatment. Although clinical signs of *R. rickettsii* infection in dogs have been described, published reports usually include descriptions of either advanced clinical cases or experimental infections caused by needle-inoculation of cultured pathogen rather than by tick bite. The natural progression of a tick-borne *R. rickettsii* infection has not been studied in sufficient detail. Here, we provide a detailed description of clinical, hematological, molecular, and serological dynamics of RMSF in domestic dogs from the day of experimental exposure to infected ticks through recovery. Presented data indicate that neither the height/duration of fever nor detection of rickettsial DNA in dogs' blood by PCR are good indicators for clinical prognosis. Only the apex and subsequent subsidence of neutrophilia seem to mark the beginning of recovery and allow predicting a favorable outcome in *Rickettsia*-infected dogs, even despite the continuing persistence of mucosal petechiae and skin rash. On the other hand the appropriate (doxycycline) antibiotic therapy of sufficient duration is crucial in prevention of RMSF relapses in dogs.

## Introduction

*Rickettsia rickettsii* is a tick-borne pathogen that causes Rocky Mountain spotted fever (RMSF) and Brazilian spotted fever in North, Central, and South America. In the United States, the American dog tick *Dermacentor variabilis* is one of the primary vectors of this pathogen. *Rickettsia* spp. seroprevalence in domestic and stray dogs in disease-endemic regions can be high – up to 68–81% [1], [2], and proximity to seropositive dogs is a risk factor for RMSF in humans [3]–[6]. Dogs themselves are susceptible to infection and clinical cases, sometimes fatal, have been described [7]–[14]. Because of their susceptibility to *R. rickettsii* and relatively high rates of tick exposure, dogs may serve as sentinels of risk for RMSF in people [3], [15]–[17]. The drug of choice for treating RMSF in dogs, as in humans, is doxycycline with the recommended treatment regimens of either 5–10 mg/kg/day for 10–21 days [18], or 10–20 mg/kg twice/day for 1 week [19] regardless of the age of the dog. Either regimen is reported to lead to quick subsidence of a fever and complete recovery with no expected sequelae or relapses.

Although clinical signs of *R. rickettsii* infection in dogs have been described, the majority of published reports present descriptions of either advanced clinical cases [9], [12], [20]–[22], or experimental infections caused by needle-inoculation of cultured pathogen rather than by tick bite [23]–[26]. The natural progression of a *R. rickettsii* infection in a canine model following exposure to infected *D. variabilis* has not been studied in sufficient detail, especially prior to the onset of evident clinical signs.

Here, we describe the progression of clinical disease via hematological, molecular, and serological data in dogs experimentally infected with *R. rickettsii* via tick bite. These observations encompassed the periods of incubation, clinical illness, treatment, and recovery. We also report a relapse of RMSF in one dog following a 2-week treatment with doxycycline.

## Results

Infected ticks placed on dogs engorged normally and dropped off within time limits expected for *D. variabilis* – 4 days for larvae, 5–6 days for nymphs, and 7–10 days for adult females [27]. Overall, 76 (22.4% of the placed) larvae and 56 (74.7%) nymphs successfully fed to repletion on dogs 181 and 424 respectively. Out the total of 34 adult *D. variabilis* placed on dogs 362 and 664, 33 (97%) ticks were collected after feeding. All 20 female ticks (10 per dog) completed their engorgement; plus 6 and 7 male ticks were collected from dogs 362 and 664 respectively at the end of the infestation (one dead male tick was removed on day 7).

### Clinical signs

**Figure 1:**
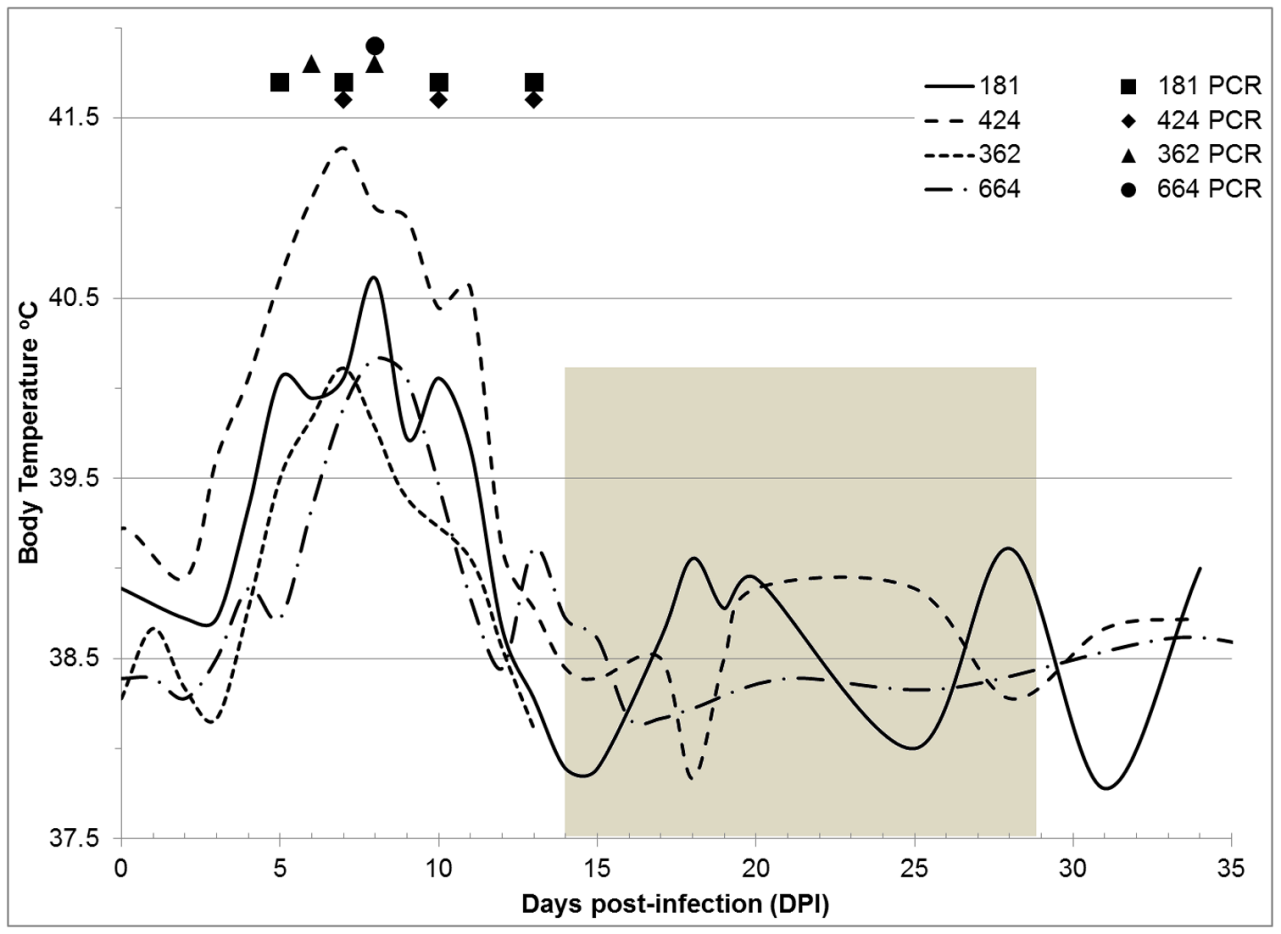
Temperature charts and detection of pathogen DNA in venous blood in dogs infected with *Rickettsia rickettsii*. (Shaded area marks the period of antibiotic treatment in dogs 424 and 664).

Body temperature charts and a summary of clinical signs observed in dogs following exposure to *R. rickettsii*-infected ticks are presented in Fig. 1 and Table 1. An increase of body temperature above 39.5°C was the earliest sign of infection in all dogs. It was recorded on the 3rd day post-infestation (DPI) in one dog, 5 DPI – in two, and 7 DPI – in one, and lasted continuously for three to nine days (Fig.1). Regardless of the time of fever onset in individual dogs, the highest temperatures (40.1 to 41.3°C) were observed at 7–8 DPI. In the two younger dogs (4 months old), febrile response to infection appeared earlier and lasted longer than in the older ones (9 months old). Following the peak of fever, the body temperature in each dog gradually declined, and by 10–12 DPI dropped to or below the pre-exposure levels. After the subsidence of fever, body temperatures continued to fluctuate (±1.5°C) throughout the period of treatment and recovery in both the antibiotic-treated (424 and 664) and the untreated (181) dogs before permanently returning to the normal – pre-exposure levels. The fall of body temperature, however, did not indicate recovery or alleviation of other clinical signs.

**Table 1:** Summary of clinical signs in dogs following infestation with *Rickettsia rickettsii–*infected *Dermacentor variabilis* ticks; (Days post-infestation).

Dog (age)	Tick life stage	Fever above 39.5°C	Depression	Lethargy	Decreased appetite	Tremors	Petechiae on ocular mucosa	Petechiae on oral mucosa	Skin rash	PCR +
181 (4 mo.)	Larvae	5–11	5–17	9–11	6–11	8–11	8–17	7–11	7–13	5–13
424^b^ (4 mo.)	Nymphs	3–11	5–17	none	6–17	6–15	6–17	11–13	6–15	7–13
362^a^ (9 mo.)	Adults	5–8	7–14^a^	8–14^a^	8–14^a^	7–8, 13–14^a^	11–14^a^	11–14^a^	8–11	6–8
664^b^ (9 mo.)	Adults	7–9	7–14	8–13	8–11, 14–15	7–14	8–18	8–17	8–17	8

a Dog 362 was euthanized at 14 DPI.

b Dogs 424 and 664 were treated with doxycycline starting 14 DPI.

Within one or two days after the appearance of fever, dogs became depressed, showing less interest in interaction or play and spending most of their time resting. This depressed state lasted for approximately a week – beyond the subsidence of fever – and in three dogs progressed into either continuous or intermittent lethargic states (Table 1). Beginning 6–8 DPI, all dogs exhibited a decrease in appetite that lasted from 6–7 to 12 days; with a total refusal of food for one or two days coinciding with the peak of lethargy. Tremors of the head, limbs, and body also appeared within 6–8 DPI. Appetite returned to normal and tremors subsided either on their own in the untreated dog (11 DPI), or within 24–72 hours after initiation of the antibiotic regimen (Table 1).

**Figure 2:**
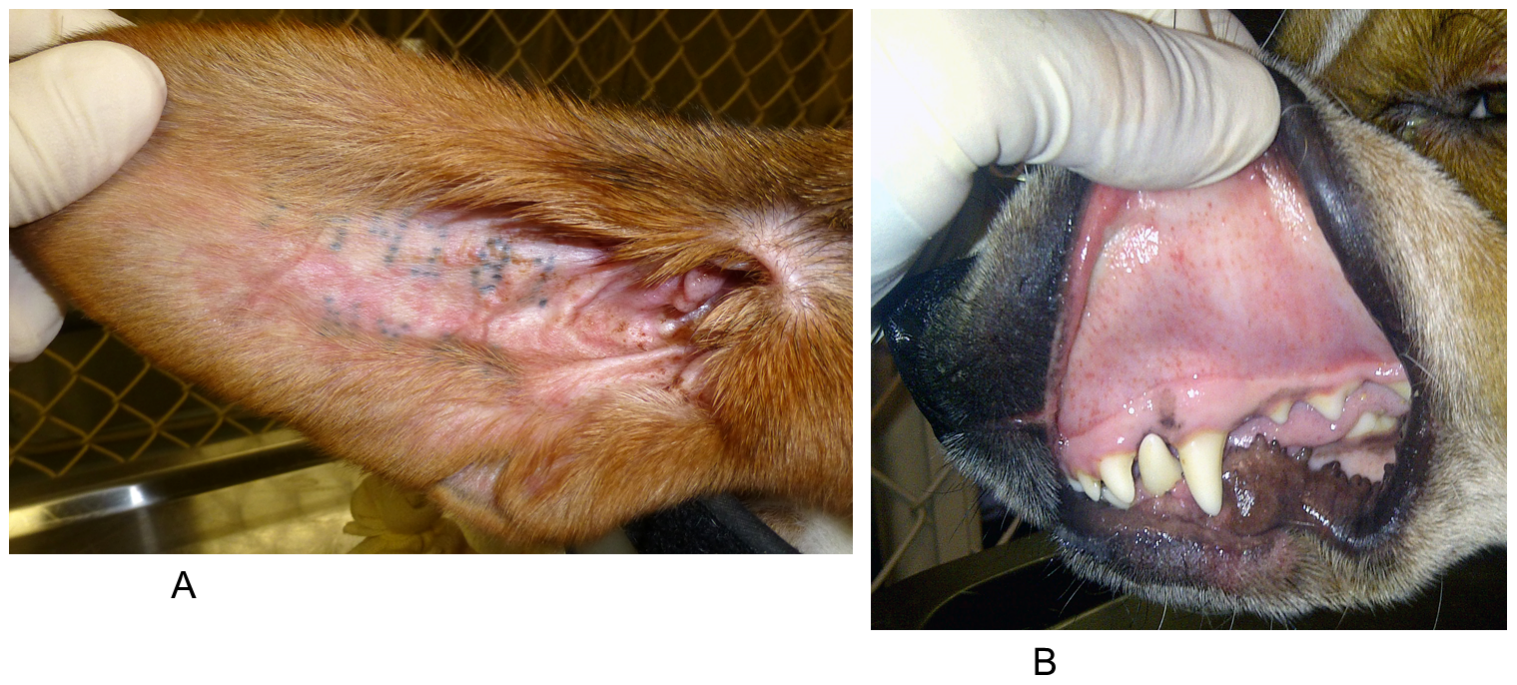
Rash in dogs infected with *Rickettsia rickettsii*. **A**– maculopapular rash on the ear of dog 181 at 11 days postinfection (DPI), **B** – petechiae on oral mucosa of dog 362 at 12 DPI.

Widespread abundant petechiae on gums and buccal mucosa, as well as macular skin rash, became noticeable 6–11 DPI and lasted well into the period of recovery or treatment (Table 1). Vascular injection of the sclera with petechiae in the conjunctiva appeared in both eyes. Extensive maculopapular rash was observed in exposed areas of the skin starting with ears and spreading to the trunk and limbs (Fig. 2).

In addition to the above signs of infection, dog 181 exhibited slightly swollen testes on 6 DPI and crusty eyes on 14 DPI. Dog 424 had clear nasal discharge on 8–13 DPI and crusty eyes on 11–13 DPI. Only one of the four dogs – 362 - appeared dehydrated on 7–12 DPI, even though it retained its body weight; it also displayed excessive salivation and scrotal edema on 7 and 12 DPI respectively. Dog 664 had excessive salivation (7 DPI), slight scrotal edema (12 DPI), and hematochezia (13–14 DPI).

Although fever in dogs 181 and 424 was higher and longer than in 362 and 664, they developed a less severe illness overall. Dogs 362 and 664 had more pronounced, acute, and longer lasting clinical signs, according to the daily overall health assessments. It is noteworthy that the less severely ill dogs were approximately five months younger. Differences in disease severity also corresponded with the fact that dogs 362 and 664 were infested with adult ticks and potentially received higher infectious doses than those infested with either infected larvae or nymphs.

### Hematological evaluation

**Figure 3:**
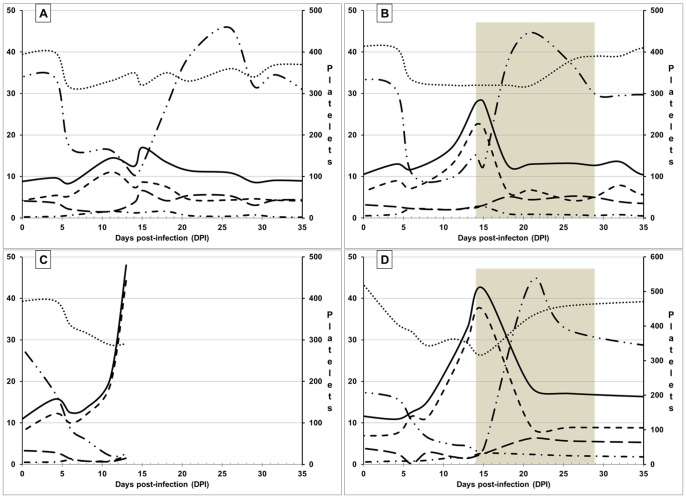
Hematologic evaluation of dogs infected with *Rickettsia rickettsii* by *Dermacentor variabilis* ticks. **A**– dog 181, **B** – dog 424, **C** – dog 362 (euthanized for pathological evaluation at 14 DPI), **D** – dog 664: •••••••••• Hematocrit value (%); ——— White blood cells (x10^3^/µl); - - - - - Granulocytes (x10^3^/µl); – – – – Lymphocytes (x10^3^/µl); – • – • – • Monocytes (x10^3^/µl); – •• – •• – •• Platelets (x10^3^/µl). Shaded areas mark periods of antibiotic treatment in dogs 424 and 664.

Significant changes in differential blood counts were observed in all experimental animals with the most prominent findings being normocytic, normochromic anemia and severe thrombocytopenia. Thrombocytopenia lasted for approximately 10 days and was followed by secondary thrombocytosis during the third and fourth weeks postinfection (Fig. 3 A–D). Platelet counts gradually returned to the pre-infection levels by 28–30 DPI in one antibiotic-treated dog (424, Fig. 3 B) as well as in the untreated dog (181, Fig. 3 A). Conversely, in the second dog treated with doxycycline, platelet numbers began decreasing within a week after the initiation of treatment, but remained higher than the pre-infection level for extended period of time (664, Fig. 3 D). Lymphocytopenia also developed in all dogs within 5 days after placement of infected ticks and was followed by lymphocytosis. Lymphocyte counts returned to normal levels by 30–35 DPI in one antibiotic-treated dog (424) and the untreated dog (181), but remained significantly elevated in the other antibiotic-treated dog (664).

All four dogs developed marked monocytosis from 4–7 DPI. Monocyte counts remained above normal for at least 2 weeks regardless of the treatment history (Fig. 3 A–D). Again, one of the two antibiotic-treated dogs (664) retained high monocyte levels well beyond the 2-wk treatment period. Three dogs - 424, 362, and 664 – also developed significant granulocytosis, which dissipated in the antibiotic-treated dogs within the first week of treatment. Eosinophil and basophil counts remained within normal limits for all dogs throughout the study. The overall changes in white blood cell counts resulted in leukocytosis in three dogs (Fig. 3 A–D).

### PCR and Serology

Rickettsial DNA was detected in the peripheral blood of the dogs on one to four occasions between 5 and 13 DPI (Fig. 1). PCR-positive blood samples generally coincided with the period of fever, with the exception of samples collected from dogs 181 and 424 at 13 DPI, after the sharp decline of body temperature. As with the febrile reaction, the duration of detectable rickettsemia in 4 month old dogs (181 and 424) was somewhat longer than in the 9 month old (362 and 664), but was inconsistent with the severity of clinical symptoms (Fig. 1, Table 1). In the older dogs, the last appearance of rickettsial DNA in peripheral blood was recorded at 8 DPI, prior to exacerbation of clinical symptoms and approximately a week before administration of doxycycline. Conversely, in the younger dogs – 181 (untreated) and 424 (treated), rickettsial DNA was detectable not only throughout the clinical illness, but after the most severe manifestation of infection had subsided. Therefore, the apparent ending of detectable rickettsemia could not be explained by either initiation of antibiotic treatment or spontaneous recovery from infection.

**Figure 4:**
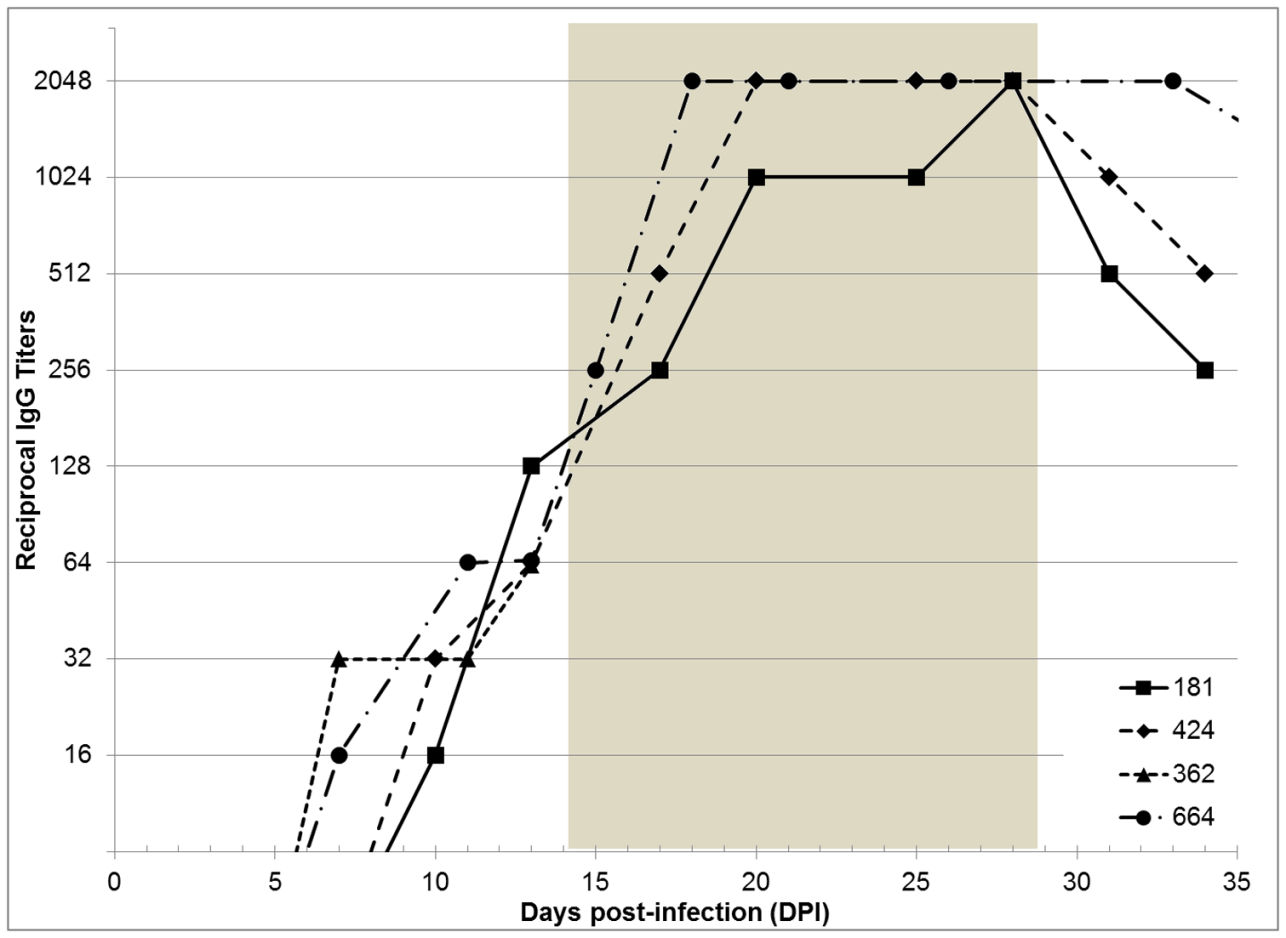
IgG antibody titers in dogs infected with *Rickettsia rickettsii*. Shaded area marks the period of antibiotic treatment in dogs 424 and 664.

All dogs seroconverted within 10–13 DPI. In the three dogs whose immune responses were monitored throughout treatment and recovery, reciprocal titers of IgG antibodies peaked at 2048 by 17–28 DPI (Fig. 4). In a dog with a more severe illness (664), the peak titers were recorded earlier than in the one with the least severe clinical signs (181). Noteworthy, antibody titers continued to rise after the subsidence of clinical signs in both treated and untreated dogs.

### Pathology evaluation

**Figure 5:**
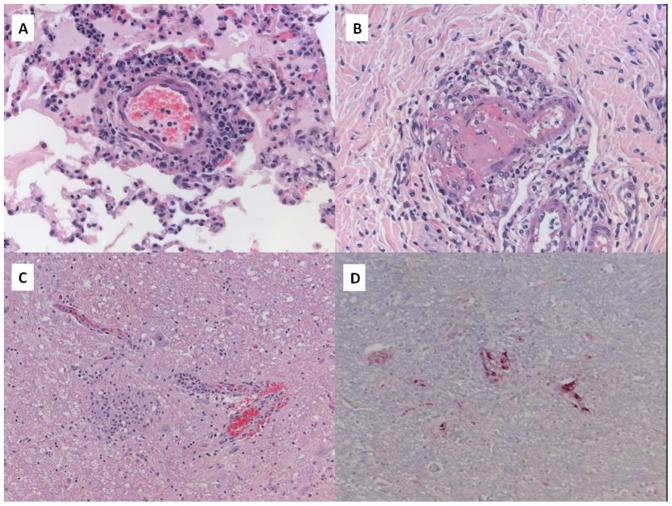
Histopathological and immunohistochemical evaluation in a dog with severe Rocky Mountain spotted fever. **A** - Canine lung with lymphohistiocytic and neutrophilic vasculitis and interstitial leukocytosis (hematoxylin and eosin staining; original magnification ×400). **B** - Canine colon, submucosa with vasculitis with thrombosis (hematoxylin and eosin staining; original magnification ×400). **C** - Canine brainstem with nonsuppurative perivascular infiltrate and glial nodule (hematoxylin and eosin staining; original magnification ×400). **D** - Canine brainstem; Immunostaining of spotted fever group rickettsial antigens in multiple vessels, immunoalkaline phosphatase staining, naphthol fast red substrate with hematoxylin counterstain (original magnification ×400).

At 14 DPI, dog 362 exhibited convulsions and was euthanized. Pathologic evaluation revealed multisystem involvement typical of acute rickettsial infection. Pertinent gross findings included pulmonary, gastrointestinal, and urinary bladder hemorrhage, and diffuse lymphadenopathy. Histopathological findings included widespread lymphohistiocytic and, to a lesser extent, neutrophilic vasculitis in multiple tissues, including: heart, lung, urinary bladder, testicle, and all examined segments of the gastrointestinal tract (Fig. 5A and B). Fibrin thrombi were seen in urinary bladder and gastrointestinal tissues (Fig. 5B). Hepatic sinusoids and pulmonary alveolar walls contained increased leukocytes throughout. Examination of brain revealed meningoencephalitis with nonsuppurative perivascular infiltrates and scattered glial nodules involving cerebrum, cerebellum, and brainstem (Fig. 5C). Mild extramedullary hematopoiesis was present in the spleen, and kidney showed minimal lymphoplasmacytic interstitial inflammatory infiltrates.

Immunohistochemical (IHC) testing for spotted fever group rickettsiae was performed on tissue specimens from brain, heart, lung, spleen, small intestine, and colon. An immunoalkaline phosphatase technique with a polyclonal anti-*R. rickettsii* antiserum was used, with appropriate positive and negative controls. Spotted fever group rickettsiae were detected by IHC in all evaluated tissues (Fig. 5D).

### Antibiotic treatment and relapse

At 14 DPI, dogs 424 and 664 were placed on pharmaceutical treatment, which included doxycycline (Vibramycin calcium, oral syrup; Pfizer Labs, Inc. New York, NY) at 5 mg/kg orally twice a day for 14 days; meloxicam (Metacam oral suspension; Boehringer Ingelheim Vetmedica, Inc St. Joseph, MO) at 0.2 mg/kg orally once a day then 0.1 mg/kg for the next two days; and Vitamin K-1 injectable (K-Ject; Butler Schein Animal Health Dublin, OH) at 2 mg/kg subcutaneous injection once a day then 0.25 mg/kg subcutaneous injections for the next two days. Due to miscommunication during a weekend, the treatment with doxycycline was maintained for a total of 16 days – two days longer than originally prescribed.

At the start of antibiotic treatment, both dogs were lethargic, anorexic, and had petechiae on the gums; extensive maculopapular rash was observed in exposed areas of the skin on the trunk and limbs, but neither dog had a fever by this time point (Table 1; Fig. 1). In addition, dog 664 appeared lethargic, reluctant to stand, and had a mild ambulatory ataxia. A complete blood count (CBC) cell differential showed an acute thrombocytopenia, leukocytosis, neutrophilia, monocytosis, and low hematocrit values in both dogs (Fig. 2).

Within 24–48 hours after the start of the treatment regimen, the tremors and anorexia resolved; the dogs became more active and began eating normal food. Scleral blood vessel injection, as well as petechiae of the oral mucosa and the rash on the skin, gradually faded away by 11–18 DPI with no apparent correlation to either antibiotic treatment or the severity of other signs (Table 1).

Platelet counts not only returned to normal, but surpassed the upper limit of the pre-infection range (Fig. 2). Total white blood cell counts as well as differential counts of granulocytes and monocytes began declining immediately after initiation of treatment. However, the reversal of neither the thrombocytopenia, granulocytosis nor monocytosis at the start of treatment could not be attributed solely to the effects of the antibiotic as exactly the same dynamics were observed in the untreated dog 181 (Fig. 2). By the end of the 16-day course of treatment, all clinical signs of infection completely resolved in both treated dogs. The dogs' body temperature, appetite, demeanor, defecation and urination remained normal. Blood-PCR remained negative throughout the treatment. Both the total and differential white blood cell counts levelled off after approximately 5–7 days after initiation of treatment and remained virtually unchanged from then on (Fig. 2).

**Figure 6:**
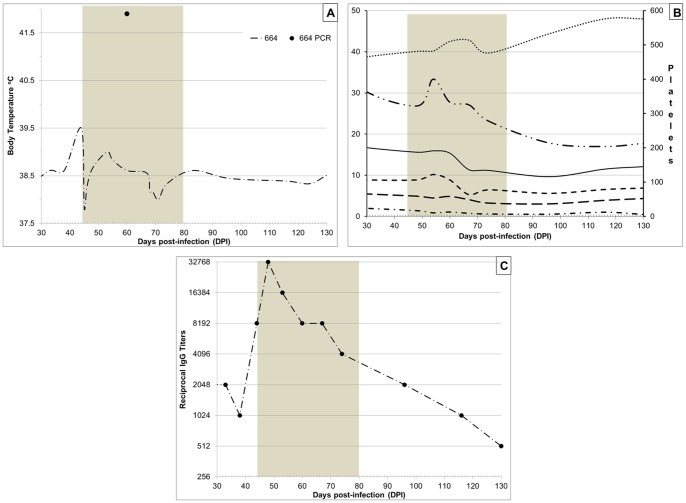
Temperature (A), Complete blood count cell differential (B): •••••••••• Hematocrit value (%); ——— White blood cells (x10^3^/µl); - - - - - Granulocytes (x10^3^/µl); – – – – Lymphocytes (x10^3^/µl); – • – • – • Monocytes (x10^3^/µl); – •• – •• – •• Platelets (x10^3^/µl), and IgG antibody titers (C) in a dog 664 during a relapse of *Rickettsia rickettsii* infection. Shaded area marks the period of antibiotic re-treatment.

**Figure 7:**
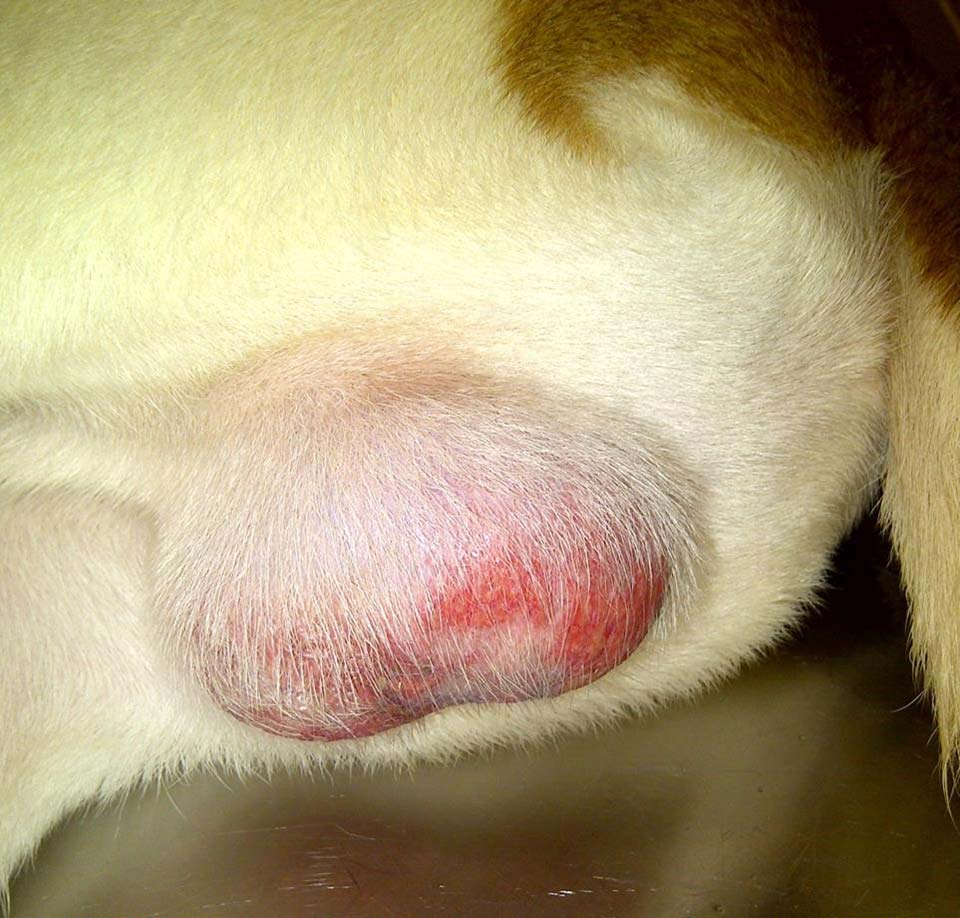
Bilateral testicular inflammation developed two weeks after the completion of a 16-day doxycycline treatment.

It was noted that in one of the dogs (664) lymphocytes, monocytes, and especially granulocytes remained elevated above the canine reference levels as well as above the pre-infection values. In view of the completely resolved clinical signs, these abnormal differential counts were not considered attributable to the pathogen persistence. However, at 44 DPI - two weeks after the completion of doxycycline treatment, this dog (664) suddenly developed severe scrotal inflammation and the core body temperature increased to 39.5°C (Fig. 6A). The dog presented as anorexic and restless. Reappearance of sparse petechiae on the gums and macular rash on exposed skin of the trunk was noted, but there was no scleral injection or conjunctivitis. Bilateral testicular edema was accompanied by acute moist dermatitis of the scrotal skin (Fig. 7). Upon palpation, testes appeared swollen and tender suggestive of underlying orchitis. The blood-PCR at this time was negative, but IgG titers against *R. rickettsii* antigen abruptly increased to 8,192 (Fig. 6C).

The dog was immediately prescribed doxycycline syrup at 5 mg/kg twice a day for a total of 27 days; enrofloxacin injectable (Baytril 2.27% solution; Bayer HealthCare, Animal Health Division, Monheim, Germany) at 3 mg/kg intramuscularly twice a day for 7 days; meloxicam oral suspension at 0.2 mg/kg once a day then 0.1 mg/kg for the next two days; and a topical triple antibiotic ointment twice a day on the affected scrotal area until resolution of the dermatitis.

Enrofloxacin (Baytril) can potentially cause damage to joint cartilage in dogs less than 8 months of age. Considering the age of dog 664 (10 months) at the time of the episode and the severity of clinical presentation, bactericidal qualities of enrofloxacin were judged to outweigh potential side effects. Meloxicam was considered the best choice among non-steroidal anti-inflammatory analgesics as it has lower potential for gastrointestinal side effects and does not impair the platelet function. Vitamin K was administered to assist in blood coagulation.

As in the previous round of antibiotic treatment, the body temperature returned to the normal range within 24 hours after the first dose of doxycycline (Fig. 6A); petechiae on oral mucosa decreased and faded within 72 hours. Conversely, the testicular inflammation persisted for over 2 weeks; it completely resolved only by 59 DPI – after 25 days of the antibiotic. Moreover, a blood sample collected at 60 DPI (16 days after initiation of treatment) was PCR-positive for rickettsial DNA. CBC differential analysis showed a gradual rise in hematocrit values and a decline in white blood cell counts to within the reference ranges. Serum IgG titers reached 32,768 by 50 DPI and then gradually declined throughout the treatment period and the following two months of observation (Fig. 6C). Together, the recurrence of rash on the oral mucosa and skin, a significant boost in anti-rickettsial antibody titers, and the reappearance of rickettsial DNA in the dog's blood strongly suggested that the testicular edema and erythema were due to a relapse of rickettsial infection.

However, because the scrotal edema and dermatitis persisted for 15 days despite intensive antibiotic treatment, the possibility of alternate causality was considered and samples of serum and urine were submitted for additional analyses. Serological testing ruled out infections with *Anaplasma phagocytophilum, Borrelia burgdorferi, Babesia microti, Bartonella henselae, B. vinsonii, Ehrlichia canis, E. ewingii, E. chaffeensis*, and canine distemper virus as a cause of testicular edema in this dog. Urine culture did not recover either aerobic or anaerobic bacteria after 96 hours of observation, and results of urinalysis were unremarkable. The negative results generated in all additional testing supported a conclusion that the orchitis, as well as the accompanying fever, were caused by a relapse of *R. rickettsii* infection and not by an alternative pathogen.

The second round of antibiotic treatment in dog 664 was concluded at 71 DPI. By that time, all clinical and hematological abnormalities had completely resolved and dog remained healthy during the additional 8.5 weeks of observation. The acute moist dermatitis resolved in parallel with the scrotal edema. Serum IgG titers gradually decreased to 512 by 130 DPI and all hematological values have remained within the normal limits (Fig. 6). No complications or sequelae were noted in dogs 181 and 424 after their recovery from the original illness up to 130 DPI (data not shown).

## Discussion

Rocky Mountain spotted fever (RMSF) is a tick-borne disease caused by *R. rickettsii*. It is a potentially fatal human illness in North and South America. Dogs infested with vector tick species are also in danger of acquiring rickettsial infection, and the prevalence of antibodies to the spotted fever group *Rickettsia* spp. in domestic and stray dogs in disease-endemic regions can exceed 60% or more [1], [2]. Moreover, proximity to seropositive dogs is a risk factor for RMSF in humans [3]–[6]. Different dog breeds vary in susceptibility to *R. rickettsii*, but the most prominent clinical signs reported in fulminant cases of canine RMSF include fever, lethargy, anorexia, anemia, thrombocytopenia, and ocular lesions [8]. In dogs with advanced stages of RMSF, and especially those experimentally infected via needle-inoculation of *R. rickettsii*, edema is often noticed in extremities but may also involve the lips, pinna of ears, penile sheath, and scrotum [28].

In the present study, all dogs exposed to either larval, nymphal, or adult *D. variabilis* ticks infected with *R. rickettsii* became clinically ill, demonstrating that immature *D. variabilis* are capable of transmitting rickettsiae to the host in amounts sufficient to cause pronounced canine RMSF, although with a varying degrees of severity. The clinical picture of canine RMSF reported in this study was similar to the previously described infections resulting from needle-inoculation of North American isolates, with exceptions of discernible edema in joints or the face, and splenomegaly, neither of which were observed here. These inconsistencies may be due to either differences in the inoculation routes and the infectious dose, the pathogen strains, or breeds of dogs used in different studies. Wide variability between strains of *R. rickettsii* in their pathogenicity for dogs is exemplified by comparing severe clinical signs observed in this study to the relatively mild illness without either hemorrhagic or neurological abnormalities in dogs infested with ticks carrying a Brazilian strain of this pathogen [7].

The most prominent signs of rickettsial infection in dogs in our study were fever, lethargy, anorexia, ocular lesions, tremors, skin rash, combined with thrombocytopenia and leukocytosis. As in previously published reports, fever above 39.5°C was the earliest sign of infection, appearing 3–7 days after tick bite. Concomitantly with fever, dogs developed monocytosis. The fever was followed by depression, anorexia, and widespread skin and mucosal hemorrhages. Thrombocytopenia, as well an abrupt decline in hematocrit values, became obvious in all dogs starting 5 DPI. Neurological signs included tremors, which began at 6–8 DPI. Leukocytosis and granulocytosis developed by 7–10 DPI, at approximately the same time as detectable titers of anti-rickettsial IgG. Dogs infected with *R. rickettsii* via tick bite did develop lymphocytopenia starting 5 DPI, but due to pronounced monocytosis and granulocytosis the total white blood cell counts increased dramatically; there was no leucopenia, which had been observed in at least one study of RMSF needle-inoculated dogs [24]. Notably, neither the height nor length of fever correlated with the severity of the illness in dogs. Moreover, in dogs with more manifest and lengthy pyrexia, other clinical signs of infection were less sever. A somewhat similar observation was made in a comparative study by E. Piranda and colleagues where needle-inoculated dogs had longer fever periods but developed less severe illness than those infected by ticks [7].

In this study, all dogs developed petechiae on both ocular and oral mucosa within 6–11 DPI after exposure to ticks infected with *R. rickettsii*. Mucosal petechiae persisted well into the periods of treatment and recovery. High frequency of ocular involvement in canine cases of RMSF in the US had previously led to a suggestion that ophthalmologic evaluation can be a useful tool in early diagnosis of canine RMSF in clinical settings [11]. Brazilian spotted fever, on the other hand, seems to very rarely cause ocular lesions in dogs [7], [14]. Whether this incongruence in clinical presentation is due to differences between *R. rickettsii* isolates, or between the predominant dog breeds used remains to be investigated.

Detectable levels of rickettsial DNA in the blood are often listed among the prominent findings associated with canine RMSF. In needle-inoculated dogs, rickettsial DNA could be detected in 50% to 100% of samples collected during the expected period of rickettsemia [7], [26]. In the present study, PCR of venous blood appeared to be less sensitive as a diagnostic tool in dogs infected via tick bites, and varied from a single positive result to repeated occurrence over a 9-day period. Differences in the duration of positive blood-PCR results between dogs could be due to either the true length of rickettsemia or varying amounts of the target DNA in the blood of different dogs, which in turn is influenced by yet undetermined factors. Although the majority of positive blood-PCR results were recorded within the fever period in individual dogs, the beginning or ending of detectable rickettsemia did not coincide with the beginning or ending of fever. These results concur with observations in dogs tick-infected with the Taiaçu strain of *R. rickettsii* from Brazil [7]. Moreover, there was no apparent relationship between the presence of detectable rickettsial DNA in dog blood and either the severity of illness or the height of IgG antibody response. Experimental inoculation studies had previously demonstrated that the severity of clinical RMSF in dogs may be related to the infectious dose [23], [24]. Our results seem to support that conclusion since dogs infested with *R. rickettsii-*infected adult ticks developed more severe illness than those infected by immature ticks, even though duration of fever and of detectable rickettsemia showed the opposite trend.

The recommended regimen for treating RMSF in dogs usually includes administration of doxycycline for one to three weeks depending on the dosage [18], [29], [30]. This regimen typically leads to quick defervescence and complete recovery with no reported sequelae or relapses. In the two dogs treated with antibiotics (424 and 664) from 14 DPI, we observed rapid improvement of attitude and appetite within 24–36 hours after administration of the first dose. Improvement in hematological values, on the other hand, could not be unequivocally attributed to the effects of the antibiotic. The 15 DPI marked the end of thrombocytopenia in both the treated and untreated (181) dogs, although the thrombocytosis developed more quickly in the treated dogs. The 14–15 day postinfection also marked the apex of leukocytosis in both the treated and untreated dogs. The only hematological value that appeared to coincide with the lessening of general signs and the recovery was the neutrophil count; granulocytosis peaked at 11–12 DPI in the dog 181, which began recovering at that point, and at 14 DPI in the antibiotic-treated dogs.

It is generally assumed that relapse does not occur in human patients who have recovered from RMSF, but relapses of other spotted fever group infections have been reported [31], [32], particularly when inappropriate antimicrobial drugs are used [33]. On the other hand, the duration of bacteriostatic (for example tetracyclines) therapy must be sufficient to allow cellular and humoral defense mechanisms to eradicate the bacteria. Although doxycycline is highly effective against rickettsiae, treatment of insufficient duration may also result in a relapse [34].

In our case, reoccurrence of fever in a dog two weeks after the conclusion of treatment was accompanied by severe testicular edema and the return of oral petechial lesions and skin rash. These also coincided with a significant (x32) rise in the titers of anti-rickettsial antibodies and a reappearance of rickettsial DNA in the venous blood. As a variety of alternate possible causes of orchitis and skin rash were ruled out by additional testing, it is concluded that this clinical episode was due to a relapse of *R. rickettsii* infection. Orchitis is rarely listed as a sign of a tick-transmitted *R. rickettsii* infection in dogs but rather associated with experimental needle-derived infections [35]. In this case, severe swelling of testes apparently resulted from relapse of RMSF transmitted to a dog by infected ticks.

Return of fevers in *R. rickettsii*-infected dogs a few days after termination of doxycycline treatment had been reported in at least one other study [26]. Elevated rectal temperatures were accompanied by a drop in attitudinal scores, but resolved in approximately a week on their own without any additional treatment. In addition, *R. rickettsii* DNA could still be detected in the blood of some of these needle-inoculated dogs on day 21 post-infection and 9 to 13 days after conclusion of the antibiotic treatment, even though authors were unable to isolate viable rickettsiae in cell culture at that point [26]. Considering that antibiotics were administered in that study for only three to seven days, these late positive blood-PCR results could have indicated persistence of rickettsiae in dogs long after defervescence and apparent resolution of all clinical signs of infection. On the other hand, the inability to culture *Rickettsia* out of PCR-positive blood samples reaffirms that a positive PCR only shows the presence of rickettsial DNA in the blood stream, but does not prove the circulation of viable rickettsiae.

The presented case demonstrates that a severe canine RMSF infection may result in harborage of the pathogen in internal organs and tissues not easily infiltrated by either antibiotics or antibodies. The bone marrow has been suggested as one of such refugia where *Rickettsia* spp. could persist through even a prolonged treatment [8]. As antibody titers begin to decline following seemingly successful treatment with doxycycline, *R. rickettsii* may escape from these refugia to cause a relapse. This relapse may not necessarily present with the same clinical signs as the original illness.

It is noteworthy that in dog 664, lymphocytes, monocytes, and especially granulocytes remained elevated above the canine reference levels after the first round of treatment and remained elevated until the relapse. They returned to normal only during the second – 27-day long – course of doxycycline. Therefore, we recommend that differential blood-cell counts should be continuously monitored in dogs treated for RMSF, and that the treatment should continue at least until total white blood cell, neutrophil, lymphocyte, and monocyte counts return to the normal range.

Altogether, this study presents a detailed description of clinical, hematological, molecular, and serological dynamics of RMSF in domestic dogs from their exposure to infected ticks through recovery. Presented data indicate that neither the height/duration of fever nor blood-PCR results should be used for the clinical prognosis. Only the apex and subsequent subsidence of neutrophilia seem to mark the beginning of recovery and predict a favorable outcome in *Rickettsia*-infected dogs, even despite the continuing persistence of mucosal petechiae and skin rash. Also, appropriate (doxycycline) antibiotic therapy of sufficient duration is crucial in prevention of RMSF relapses.

## Materials and Methods

### Ethics Statement

The study was undertaken at a facility fully-accredited by the Association for the Assessment and Accreditation of Laboratory Animal Care (AAALAC) International. All procedures and husbandry were performed in accordance with the recommendations in the Guide for the Care and Use of Laboratory Animals 8th ed. All procedures of this study were pre-approved by the Centers for Disease Control Institutional Animal Care and Use Committee (IACUC) and monitored by a veterinarian stationed on-site. The US Animal Welfare Act and all associated regulations were strictly followed.

We used four mix-breed (hound-type) male dogs. Dogs 181 and 424 were 4 months-old, and dogs 362 and 664 were 9 months-old. Throughout the study, the dogs were housed indoors, in a climate-controlled animal facility, which precluded unintended exposure to any arthropod-borne agent including *Rickettsia* spp. The absence of antibodies to spotted fever group (SFG) rickettsiae in each dog was confirmed prior to enrollment into the study by the indirect immunofluorescence assay (IFA) as described below. Dogs were provided with water and a standard dry food diet (Laboratory Canine Diet 5006; Purina, Fairburn, GA, USA) ad libitum. The appetite, behavior, disposition, and level of activity of each dog were monitored twice daily throughout the study. Dogs exhibiting decreased appetite were fed soft canned dog food (Pedigree, McLean, VA, USA). Throughout the period of infection and treatment, the body temperature of each dog was measured every morning; and temperatures ≥39.5°C were defined as febrile. Venous blood and serum samples were collected twice weekly for the differential blood cell counts, serum chemistry panels, PCR, and serology.

*Dermacentor variabilis* ticks infected with *R. rickettsii* (isolate BSF-Di6) were derived from the second generation of our infected colony. Prior to this study, larval and nymphal ticks were acquisition fed upon guinea pigs needle-inoculated with the second Vero cell passage of isolate BSF-Di6, which was previously propagated and stored in yolk-sack culture (5 passages) [36], [37]. Laboratory tick colonies were maintained by feeding upon infected guinea pigs (*Cavia porcellus*) and specific pathogen free (SPF) New Zealand white rabbits (*Oryctolagus cuniculus*) as previously described [27].

For the experimental infection, ticks were placed inside feeding bags glued to a shaven area on the dog's back using Kamar adhesive (Kamar, Inc., Steamboat Springs, CO), which is approved for veterinary use [38]. Dogs were subjected to tick infestation levels comparable to those seen in natural setting [39], [40]: dog 181 received 250 larvae derived from the progeny of an infected female tick (25% filial prevalence of infection); dog 424 received 75 nymphs from a cohort where 64% were infected with *R. rickettsii*; dogs 362 and 664 each received 10 female and 7 male ticks from the infected colony with 90% prevalence of infection.

At two weeks post-infestation, one of the dogs (362) was euthanized. The assessment of organ pathology; tissue histology and immunohistochemistry assessments were performed as previously described [41]. Two dogs (424 and 664) were treated orally with doxycycline (Vibramycin Ca syrup, 50 mg/5 ml; Pfizer Labs, New York, NY) at 5 mg/kg twice a day starting at two weeks post-infestation and continuing for 14 days. The remaining dog181 (whose clinical signs had already began subsiding) was allowed to recover from illness on its own under continuous monitoring.

### PCR and Serology

Whole blood (200 µl) and serum (500 µl) samples from dogs were collected aseptically into either 1.7 ml microcentrifuge tubes (Corning Inc., Lowell, MA) containing 5 microliters of 2% EDTA (Sigma Aldrich, St. Louis, MO) or Microtainer serum separator tubes (Becton Dickinson and Co., Franklin Lakes, NJ). Blood samples were stored at −20°C until tested by PCR for the presence of *R. rickettsii* DNA and sera samples were refrigerated at 4°C for serologic testing.

DNA extraction and PCR procedures were carried out in separate facilities. DNA was extracted from tick and blood samples using the Qiagen DNEasy Blood & Tissue kit and Flexi Gene kit (Qiagen Inc., Valencia, CA) respectively according to manufacturer's protocols. The presence of Rickettsial DNA was detected by PCR using primers RR190-547F and RR190-701R to amplify a 154-bp fragment of the *romp*A gene of *Rickettsia* spp. as described by Eremeeva et al. [42]. Blood samples were tested in duplicate. Two negative (distill water) and two positive (*R. massiliae* plasmid) were included into each run. Samples demonstrating amplification prior to 40 cycles with appropriate amplicon melting temperature in both replicates were considered positive.

IFA was performed on dog sera, as previously described [43], using FITC labeled goat anti-dog IgG (γ) conjugate diluted per manufacturer's recommendations (KPL, Inc. Gaithersburg, Maryland, USA). Slides were spotted with *R. rickettsii* antigen, air-dried and fixed in acetone. Serum samples were initially screened at 1/16 and 1/256 dilutions, and positive samples were titrated to the endpoint in a two-fold dilution series. Serologic data are reported as the reciprocal of the last dilution showing positive fluorescence. Titers ≥32 were considered positive.
